# Prognostic Relevance of Tumor-Infiltrating Immune Cells in Cervix Squamous Cell Carcinoma

**DOI:** 10.3390/cancers15204952

**Published:** 2023-10-12

**Authors:** Carl Mathis Wild, Fabian Garrido, Christian Dannecker, Melitta B. Köpke, Marie-Christine Chateau, Florence Boissière-Michot, Helene H. Heidegger, Aurelia Vattai, Mirjana Kessler, Udo Jeschke, Vincent Cavaillès

**Affiliations:** 1 Department of Obstetrics and Gynecology, University Hospital Augsburg, Stenglinstrasse 2, 86156 Augsburg, Germany; mathis.wild@uk-augsburg.de (C.M.W.); fabian.garrido@uk-augsburg.de (F.G.); christian.dannecker@med.uni-augsburg.de (C.D.); melitta.koepke@uk-augsburg.de (M.B.K.); 2 Department of Data Management and Clinical Decision Support, Faculty of Medicine, University of Augsburg, 86159 Augsburg, Germany; 3 Translational Research Unit, Montpellier Cancer Institute Val d’Aurelle, 208 rue des Apothicaires, F-34298 Montpellier, France; marie-christine.chateau@icm.unicancer.fr (M.-C.C.); florence.boissiere@icm.unicancer.fr (F.B.-M.); 4 Department of Obstetrics and Gynecology, University Hospital, LMU Munich, Marchioninistraße 15, 81377 Munich, Germany; helene.heidegger@med.uni-muenchen.de (H.H.H.); aurelia.vattai@med.uni-muenchen.de (A.V.); mirjana.kessler@med.uni-muenchen.de (M.K.); 5 Institut de Recherche en Cancérologie de Montpellier (IRCM), INSERM U1194, Université Montpellier, CNRS, F-34298 Montpellier, France; vincent.cavailles@inserm.fr

**Keywords:** cervical cancer, tumor-infiltrating immune cells (TIICs), cervix adenocarcinoma (CAC), cervix squamous cell carcinoma (CSCC), disease-free survival (DFS), overall survival (OS)

## Abstract

**Simple Summary:**

Although tumor immune infiltration has been analyzed in a variety of tumor entities, it has never been investigated in cervical cancer in relation to histological subtypes and prognosis. Therefore, the aim of this study was to investigate the prognostic impact of tumor immune infiltration in a panel of 238 sporadic cervical cancer cases by quantifying the levels of tumor-infiltrating immune cells (TIICs) and correlate them with the histological subtype and with patient survival. The level of TIIC was significantly enhanced in cervix squamous cell carcinomas (CSCC) versus cervix adenocarcinomas (CAC) and represented an independent positive prognosticator for disease-free survival (DFS) in patients with CSCC.

**Abstract:**

There exists a variety of studies about tumor-infiltrating immune cells (TIICs) in cervical cancer, but their prognostic value in correlation with the histopathological subtype has never been investigated. Therefore, the aim of this study was to quantify TIICs in a panel of 238 sporadic cervical cancers and investigate the correlation with cervical cancer subtype and patient survival. TIICs levels were significantly increased in the subgroup of CSCC (191 samples) in comparison to CAC (47 samples). In CSCC, TIICs’ infiltration showed a negative correlation with age, FIGO stage and with the histone protein modification H3K4me3. Moreover, in CAC, it was positively correlated with p16 and with the glucocorticoid receptor and inversely correlated with the MDM2 protein and with H3K4me3. Interestingly, immune infiltration was an independent positive prognosticator for disease-free survival (DFS) in patients with CSCC, those bearing tumors with the strongest TIICs infiltration showing the better DFS. Altogether, the present study provides a differentiated overview of the relations between TIIC levels and prognosis in patients with CSCC vs. patients with CAC.

## 1. Introduction

The prognostic impact of tumor-infiltrating immune cell (TIIC) populations in cervical cancer is still debated, probably due to the fact that prognostic studies on TIICs in cervical cancer cases are limited [1]. This is surprising because the cytotoxic activities of immune cells isolated from gynecologic malignant tumors (including uterine cervical cancers) against various fresh tumor cells were reported more than 30 years ago [2].

In addition to immune cell quantification, major effort has been put into the isolation and characterization of TILs from cervical carcinomas in recent years [3]. In order to define the anticancer-directed immune response in situ, the group of Höhn et al. characterized CD4(+) and CD8(+) T cells from peripheral blood lymphocytes, freshly harvested tumor tissue and immune cells from a patient with cervical cancer [4]. The group of Santin et al. found that cervical cancer-infiltrating immune cells contain higher numbers of type 1 cytokine expressors and DR+ T cells compared with lymphocytes from tumor draining lymph nodes and peripheral blood [5].

Additional research approaches include the immune concept of human papillomaviruses and related antigens in local cancer milieu of human cervical neoplasia [6]. Immune cells in the tumor microenvironment may be functionally inhibited and lose the ability to clonally proliferate because of decreased expression of IL-2Rα [6].

A more recent study on infiltrating immune cells characterization showed that tumor-infiltrating lymphocytes in cervical cancers contain a higher proportion of FoxP3(+) T lymphocytes [7]. In addition, our own studies showed that high CCL22(+)-infiltrating cells, particularly M2-like macrophages, are associated with a poor outcome of cervical cancer patients [8]. CCL22 expression is positively correlated with FoxP3 expression [8], could polarize TAMs toward M2a macrophages [9] and may represent a novel prognostic marker and therapeutic target for the treatment of cervical cancer.

Because the link between cervical cancer histopathological subtypes, prognosis and immune infiltration is still unclear, the aim of this study was to quantify the stromal density of TIICs cells in a panel of 238 sporadic cervical cancers. Using this cohort, cells with lymphocyte or plasma cell morphology were assessed in three classes (low, moderate and strong infiltration), and we investigated the correlation with cervical cancer subtype and patient survival.

## 2. Materials and Methods

### 2.1. Tissue Sample

For this study, we included formalin fixed paraffin embedded cervical cancer samples of 238 patients (without distant metastasis) who underwent surgery in the years 1993–2002 at the Department of Gynecology and Obstetrics, Ludwig Maximilian University Munich, Germany (see Table 1 for a description of the cohort characteristics). This happened without any preselection. Only patients with tumors corresponding to CSCC and CAC histological subtypes participated in the cohort. The clinical and follow-up data, such as patient age, overall survival (OS), disease-free survival (DFS), lymph node status, tumor size, presence of metastases, histopathological grading, tumor subtype and FIGO 2009 (Fédération Internationale de Gynécologie et d’Obstétrique) stage, were retrieved from the Munich Cancer Registry.

### 2.2. Ethical Approval

The tissue samples used in this study where leftover material after all diagnostics had been completed and were retrieved from the archive of Gynecology and Obstetrics, Ludwig Maximilian University, Munich, Germany. All patients gave informed consent for additional research before undergoing surgery. The procedures were in accordance with the Helsinki Declaration of 1975. All information and data of the patients were fully anonymized and encoded for further statistical analysis. This study was approved by the Ethics Committee of the Ludwig Maximilian University, Munich, Germany.

### 2.3. Quantification of TIICs

TIICs were quantified by an experienced gynaeco-pathologist (M-C.C) following the guidelines issued by the International TIL Working Group [10]. We adapted the method in which we used the hematoxylin nuclear counterstaining of an immunohistochemical procedure published recently [11]. For the quantification of TIIC, cells that had lymphocyte or plasma cell morphology were taken into account. Only stromal TIICs were quantified, and mononuclear immune cells within tumor cell nests were excluded from the TIIC assessment. According to the amount of TIIC in the stroma, each sample was classified with low, moderate or strong infiltration as a function of the area occupied by TICCs relative to the whole stroma area.

### 2.4. Statistical Analysis

For statistical analysis, the IBM Statistical Package for the Social Sciences (IBM SPSS Statistic v24.0 Inc., Chicago, IL, USA) was used. In the case of missing data, case-by-case exclusion was used for the corresponding analysis. Spearman correlation was used to assess the correlation between TIIC levels and various stains of the same samples, including glucocorticoid receptor, E6, LCoR, RIP140, nuclear p53, H3K9ac and H3K4me3, which had recently been published [12,13,14].

Survival times were compared by Kaplan–Meier analysis. DFS was defined as time in months between initial diagnosis and local recurrence or metastasis occurring. OS was defined as time in months between initial diagnosis and death. The Cox Mantel log-rank test was used for the differences in OS. Non-parametric tests such as Kruskal–Wallis or Mann–Whitney U tests were performed for comparisons of different groups. A *p*-value < 0.05 was considered to be significant. The *p*-value and the number of patients analyzed in each group are given for each chart.

## 3. Results

### 3.1. Quantification of TIICs

**Figure 1:**
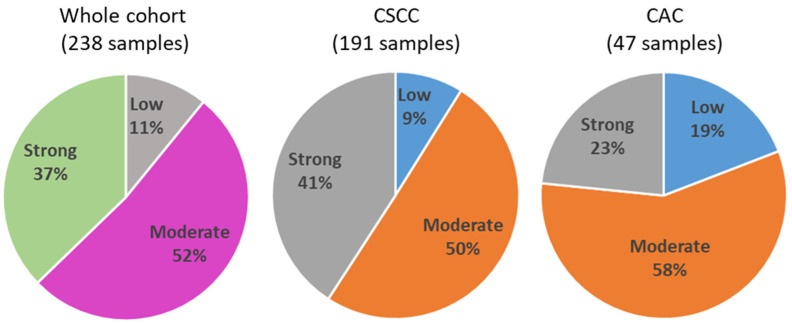
Distribution of TIICs in the whole cohort, in CSCC and in CAC tissues, expressed as percentage of cases.

Quantification of TIICs in the whole cohort (238 samples) revealed 26 cases (11%) with low levels of immune cells, 124 cases (52%) with moderate levels of immune cells and 89 cases (37%) with strong infiltration of immune cells (Figure 1). In CSCC samples (201 samples), we identified 17 cases (9%) with low, 96 cases (50%) with moderate and 78 cases (41%) with strong infiltration of immune cells. In CAC samples, we found 9 cases (19%) with low, 27 cases (58%) with moderate and 11 cases (23%) with strong infiltration of immune cells.

**Figure 2:**
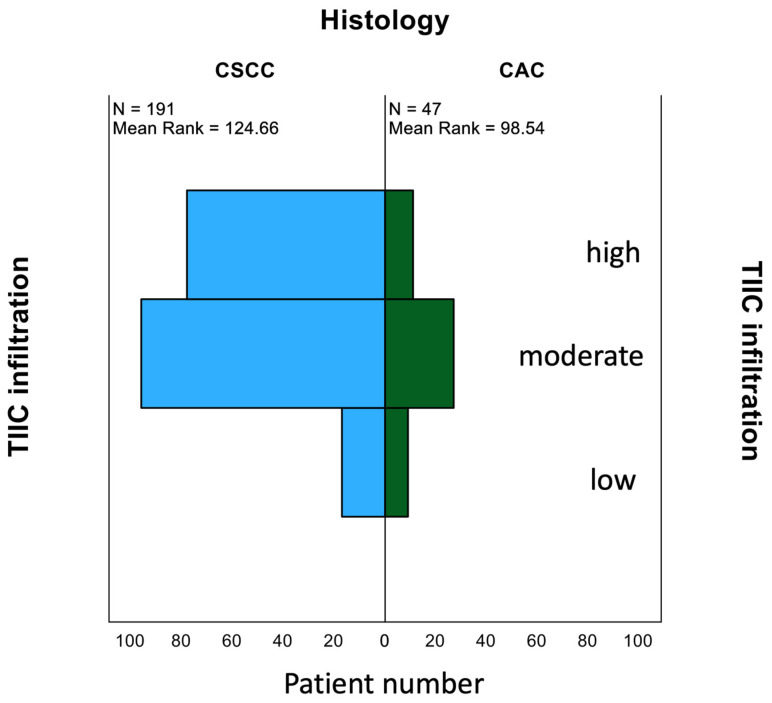
Difference in the distribution of TIICs between CSCC and CAC measured with Mann–Whitney U test (*p* = 0.01).

Infiltration of immune cells appeared significantly different according to the histological subtype of cervical cancer. Indeed, in CSCC, we identified significantly higher TIIC levels as compared to CAC (Figure 2, *p* = 0.01).

**Figure 3:**
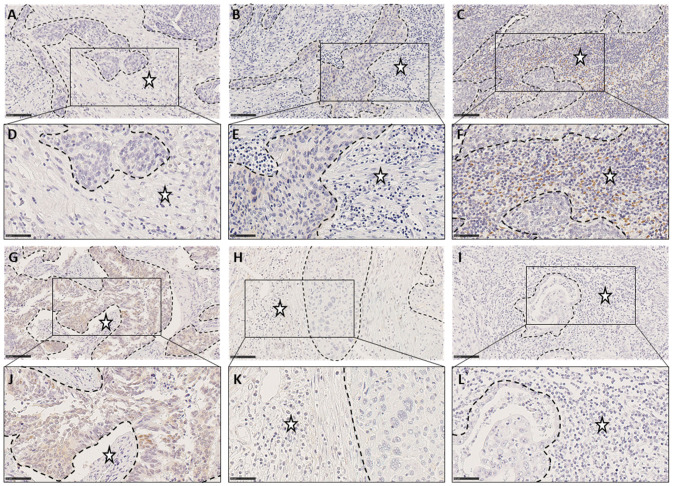
Illustration of low (**A**,**D**,**G**,**J**), moderate (**B**,**E**,**H**,**K**) or marked (**C**,**F**,**I**,**L**) inflammation in three CSCC samples (**A**–**F**) and 3 CAC (**G**–**L**). Tumor cell nests are surrounded by dotted line and stars indicate stroma. (**A**–**C** and **G**–**I** Scale bar: 100 µm; **D**–**F** and **J**–**L**, Scale bar: 50 µm).

Examples for low, moderate and strong TIIC infiltration in CSCC (panels A–F) and CAC (panels G–L) are in Figure 3.

### 3.2. Correlation Analyses of TIIC Levels with Tumor Properties and Protein Markers

We then analyzed the correlation of TIIC levels with tumor properties and protein staining results obtained from former studies of our group. Results for CSCC are presented in Table 2, showing that TIIC infiltration shows a negative correlation with age, FIGO stage and with the nuclear histone protein modification H3K4me3 [15].

The correlation between TIIC and cervical cancer prognostic markers in the CAC subtype is shown in Table 3. TIIC levels exhibited a positive correlation with p16 expression [16], a negative correlation with MDM2 expression [16] and a positive correlation with the glucocorticoid receptor [14].

### 3.3. Survival Analyses According to Histology

**Figure 4:**
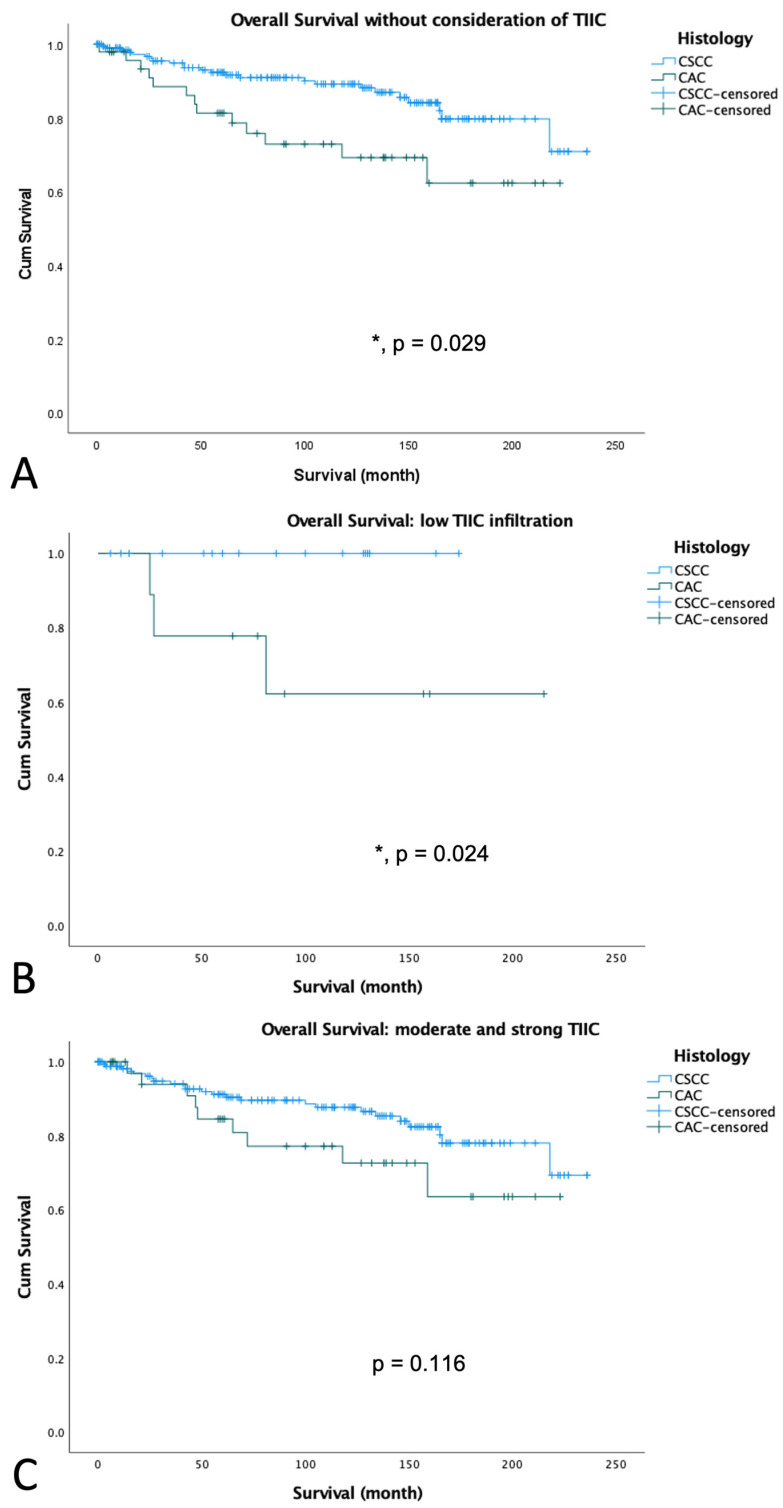
Kaplan–Meier survival analyses for OS in different histological subtypes of cervical cancer in the whole cohort (**A**), in patients with low level of TIIC (**B**) or in patients with moderate and strong TIIC (**C**). Significant differences are marked with an asterisk (*) and the exact *p*-value is added to the figure.

TIIC levels were significantly enhanced in CSCC versus CAC, as shown in Figure 2. Survival analyses of our patient cohort showed that patients with CSCC had a significantly better OS than patients with CAC (Figure 4A, *p* = 0.009; Table 4), which is in accordance with the literature [17]. Subgroup analyses including TIIC revealed that this difference is determined by the group of patients with low TIIC infiltration (Figure 4B, *p* = 0.024). Patients with moderate and strong TIIC showed no significant OS differences in CSCC or CAC (Figure 4C, *p* = 0.116). Survival analyses of our patient cohort showed that patients with CSCC had a better disease-free survival compared to patients with CAC, although the difference was at the limit of significance (Figure S1, *p* = 0.088).

### 3.4. Survival Analyses According to TIIC in Different Histological Subtypes of Cervical Cancer

**Figure 5:**
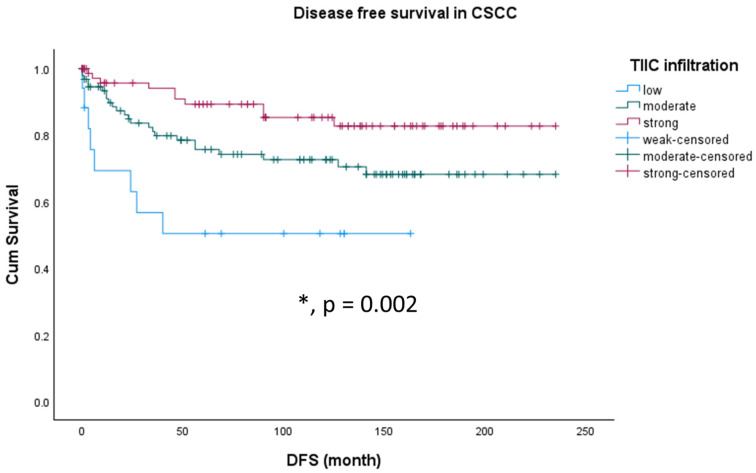
Kaplan–Meier survival analyses for disease-free survival (DFS) according to immune infiltration in CSCC cases. Significant differences are marked with an asterisk (*) and the exact *p*-value is added to the figure.

Within our cohort, we analyzed the influence of TIIC infiltration on overall survival (OS) in the different histological subgroups. Neither in CSCC nor in CAC was TIIC level a prognosticator for OS. By contrast, immune infiltration was a strong positive prognosticator for disease-free survival (DFS) in patients with CSCC. Patients with a CSCC showing low TIIC infiltration showed the shortest DFS time, while patients with strong TIIC infiltration showed the longest DFS time and patients with moderate TIIC infiltration were between both groups (Figure 5 and Table 5, *p* = 0.002). In patients with CAC, the TIIC level was without prognostic value.

Due to the discrepancy between short DFS and long OS in patients with CSCC and low TIIC (Figure S2), we compared all three groups of CSCC. There was only a significant difference between patients with CSCC and low TIIC and patients for CSCC with moderate or high TIIC for age (*p* = 0.016) but no difference for grading, pT, pN or FIGO-stage (Table S1). DFS and OS were similar in CSCC with moderate or strong immune infiltration (Figure S2).

Finally, multivariate Cox regression including TIIC infiltration, age and various tumor parameters identified TIIC and tumor size (pT) as independent prognostic factors for DFS in CSCC (Table 6).

## 4. Discussion

Within this study, we analyzed the density of TIICs in a cohort of 238 cervical cancer cases in relation to the histological subtype and patient survival. This scoring of inflammatory cells was significantly enhanced in CSCC versus CAC. Survival analyses of our patient cohort showed that patients with CSCC had a significantly better OS than patients with CAC, this difference being observed only in the group of patients with low TIIC. This is in line with Chen et al., who describe a shorter OS for patients with less intraepithelial CD8+ lymphocyte counts [18]. Interestingly, immune infiltration was an independent positive prognosticator for DFS in patients with CSCC.

Because survival rates are different in CSCC compared to CAC, we performed correlation analyses separately for both histological subtypes. In the group of CSCC, tumor infiltration by immune cells was negatively correlated with age, FIGO stage and the histone protein modification H3K4me3; the latter was analyzed in a recent study of our group [6]. H3K4 methylation is a modification that occurs at the fourth lysine residue of the N-terminus of histone H3. It can be mono-, di- and trimethylated, which makes the analysis of its effects on the genome even more complex [19,20]. H3K4me3 is generally associated with transcriptional activation and has been proposed as a predictive factor of poor prognosis in several types of cancer, such as liver and cervical cancer [15,21]. In our former analyses, high expression of H3K4me3 was associated with reduced overall and recurrence-free survival; this is in accordance with our negative correlation results with TIIC infiltration. Within this study, we found that CSCC patients with strong TIIC infiltration showed the longest DFS time.

In the group of CAC patients, tumor infiltration by immune cells showed a positive correlation with p16 [16] and with the glucocorticoid receptor (GR) [14] and a negative correlation with MDM2 [16]. The cell cycle regulation protein p16 is expressed at high levels in HPV-infected epithelial cells, which is why it acts as a marker for the diagnosis of an HPV-associated carcinoma [22,23]. The positive association of high TIIC rates and p16 expression has already been described in a variety of carcinomas, including oropharyngeal and hypopharyngeal [24,25], breast [26], oropharynx squamous cell carcinomas [27] and others, but not in cervical cancer cases and not in relation to histopathology. MDM2 promotes the ubiquitination and degradation of p53 [28]. On the one hand, p53 is regulated by MDM2 oncoprotein through a negative feedback mechanism in non-carcinoma tissue. On the other hand, there is an association between MDM2 and p53 polymorphisms and the advancement of cervical carcinoma [29]. Again, our findings are in agreement with another study on head and neck squamous cell carcinomas, showing that proliferative lymphocytes are vulnerable to MDM2 inhibition [30]. This finding might explain that high expression of MDM2 is associated with low TIIC rates in the adenocarcinoma subtype of cervical cancer. Finally, we detected a positive correlation of TIIC with GR in CAC cases. Although this relationship has not been described before, triple-negative breast cancers with expression of glucocorticoid receptor in immune cells showed a better prognosis [31]. Our former study showed the same result; an advanced GR expression went along with significantly better overall survival compared to low GR expression in cervical cancer cells [14].

It has been long known that patients with CSCC have a significantly better OS than patients with CAC [32,33], and this was also confirmed with our collection of patients. Inclusion of TIIC revealed that this effect is determined only in the group of low TIIC infiltration. In addition, in that group, no patient with CSCC and low TIIC infiltration died. Although this concerns only a small group of patients (17 out of 191; 8.9%), this subgroup can be reassured about their OS rate. 

In contrast, in CSCC cases, patients with low peritumoral inflammation showed a very short DFS time. Immune infiltration was an independent positive prognosticator for DFS in patients with CSCC. Patients with CSCC and low levels of TIIC showed the shortest DFS time whereas patients with CSCC and strong TIIC infiltration showed the longest DFS time. On first viewing, this seems to be contradictory with the OS rate of patients with low TIIC. In general, recurrence is not protective, and this was also true within our study group. We saw a strong correlation of recurrence and fatality rate in the whole cohort of squamous carcinoma cases (Correlation Coefficient = 0.451, *p* < 0.001). Therefore, only the group of CSCC patients with low TIIC seemed to be not affected by a worse outcome in combination with early recurrence. In other tumor entities, such as oral squamous carcinomas (OSCC), TILs in the nonrecurrent group were significantly higher than those in the recurrent group [34]. In addition, a high ratio of TILs was associated with an OS improvement in OSCC patients. This is in opposite to our findings on cervical cancer. On the other hand, low PD-L1 expression in TILs predicted local recurrence in oral squamous cell carcinomas [35]. Although we did not investigate PD-L1 on TIICs, this could also be an explanation for our findings.

## 5. Limitations

This study has some limitations, considering its retrospective nature and the way TIICs were assessed. For instance, we herein only performed a global analysis of TIICs, and since these cells may be immunogenic or immune-suppressive, more precise methods based on immunohistochemical detection of the different lymphocyte subtypes (including cytotoxic and regulatory T cells, or B/plasma cells) would have been more informative. These points will be addressed in further studies, which are also needed to determine the prognostic value of checkpoint molecule expression on different TIIC populations.

Another limitation is the missing data for the kind of recurrence observed (local recurrence, metastasis to local lymph nodes or distant organs). In addition, individual groups, e.g., CAC with a low TIIC level, are very small, so that a check on another cohort should be performed before generalizing the results.

## 6. Conclusions

TIIC infiltration is an independent positive prognosticator for DFS in patients with CSCC. Patients with CSCC and a low TIIC infiltration showed the shortest DFS time whereas patients with CSCC and a strong TIIC infiltration showed the longest DFS time and patients with moderate TIIC infiltration were between both groups. Neither in CSCC nor in CAC was TIIC level a prognosticator for OS. Moreover, CSCC patients with low levels of TIIC represented an atypical group of cases with early recurrence but very good outcome.

## Figures and Tables

**Table 1:** Baseline characteristics for CSCC and CAC samples.

Parameter		CSCC (n = 191)		CAC (n = 47)		
		Mean	Std. dev.	Mean	Std. dev.	*p*
Age		49.69	12.89	48.94	12.31	0.718
		Number	Percentage	Number	Percentage	
pT (summarized)	pT1	42	22.0	10	21.3	0.280
	pT2	37	19.4	14	29.8	
	pT3	112	58.6	24	48.9	
pN	pN1	76	39.8	14	29.8	0.205
	pN0	115	60.2	33	70.2	
Grading	G1	12	6.3	7	14.9	0.129
	G2	112	58.6	26	55.3	
	G3	61	31.9	12	25.5	
	Missing	6	3.1	2	4.3	
FIGO 2009	1–1B2	43	22.5	18	38.3	0.090
	2–4	74	38.7	16	34.0	
	Missing	74	38.7	13	27.7	

**Table 2:** Correlation between TIIC and cervical cancer prognostic markers in the CSCC subtype. The table shows correlation coefficient, significance (Sig.) and number of cases (N) in CSCC.

Age	Correlation Coefficient	−0.177
	Sig. (2-tailed)	**0.015**
	N	187
FIGO	Correlation Coefficient	−0.184
	Sig. (2-tailed)	**0.011**
	N	191
H3K4me3 (nuclear)	Correlation Coefficient	−0.293
	Sig. (2-tailed)	**<0.001**
	N	191

Significant differences are shown in bold.

**Table 3:** Correlation between TIIC and cervical cancer prognostic markers in the CAC subtype. The table shows correlation coefficient, significance (Sig.) and number of cases (N).

p16 (cytoplasmic)	Correlation Coefficient	0.322
	Sig. (2-tailed)	**0.031**
	N	45
MDM2 (nuclear)	Correlation Coefficient	−0.422
	Sig. (2-tailed)	**0.003**
	N	47
Glucocorticoid receptor (nuclear)	Correlation Coefficient	0.389
	Sig. (2-tailed)	**0.007**
	N	47

Significant differences are shown in bold.

**Table 4:** Mean OS time according to histology. Estimate = mean survival in months.

Histology	Mean Estimate	Std. Error	95% Confidence Interval	
			Lower Bound	Upper Bound
CSCC	204.839	5.783	193.505	216.173
CAC	169.266	12.948	143.887	194.645
Overall	199.147	5.502	188.363	209.930

**Table 5:** Mean DFS according to TIIC infiltration (low, moderate and strong) in CSCC. Estimate = mean survival in month.

Histology	TIIC	Mean Estimate	Std. Error	95% Confidence Interval	
				Lower Bound	Upper Bound
CSCC	Low	88.798	18.835	51.882	125.714
	Moderate	174.022	10.521	153.401	194.643
	Strong	204.295	8.942	186.768	221.821
	Overall	181.431	7.160	167.398	195.465

**Table 6:** Multivariate Cox regression analysis of immune infiltration and tumor parameter in relation to DFS in CSCC. pN = lymph node involvement, pM = distant metastasis, pT = tumor size.

	Coefficient	Significance	Hazard Ratio	95% Confidence Interval	
				Lower	Upper
Immune infiltration	−0.669	**0.007**	0.512	0.316	0.830
pN	0.255	0.477	10.291	0.638	2.611
pM	−0.526	0.263	0.591	0.235	1.485
age	0.008	0.520	1.008	0.983	1.034
pT	0.225	**0.016**	1.253	1.043	1.506
FIGO	−0.018	0.632	0.982	0.910	1.059
Grading	0.597	0.056	1.816	0.984	3.351

Significant differences are shown in bold.

## Data Availability

The data presented in this study are available on request from the corresponding author. The data are not publicly available due to ethical issues.

## Supplementary Materials

The following supporting information can be downloaded at: https://www.mdpi.com/article/10.3390/cancers15204952/s1, Figure S1: Kaplan–Meier survival analyses for DFS in the two different histological subtypes of cervical cancer. Figure S2: Survival analyses for DFS and OS in CCSCC with low (A), moderate (B) or strong TIIC levels (C). Table S1: Comparison for age, pT, pN, grading and FIGO 2009 in CSCC with different levels of TIIC.
