# Supplementary material for: Prognostic Relevance of Tumor-Infiltrating Immune Cells in Cervix Squamous Cell Carcinoma
Source: Cancers (Basel). 2023 Oct 12;15(20):4952. doi: 10.3390/cancers15204952 (PMC10605287; doi:10.3390/cancers15204952)
Supplement: Supplementary file 1 [file cancers-15-04952-s001.zip › cancers-2548203-supplementary.pdf]

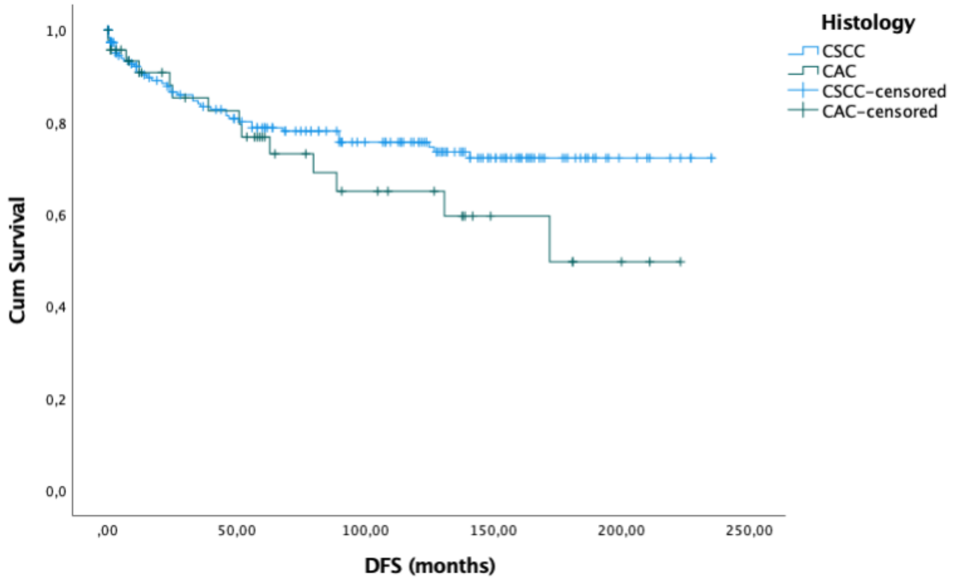

Figure S1: Kaplan-Meier survival analyses for DFS in the two different histological subtypes of cervical cancer (p=0.197)

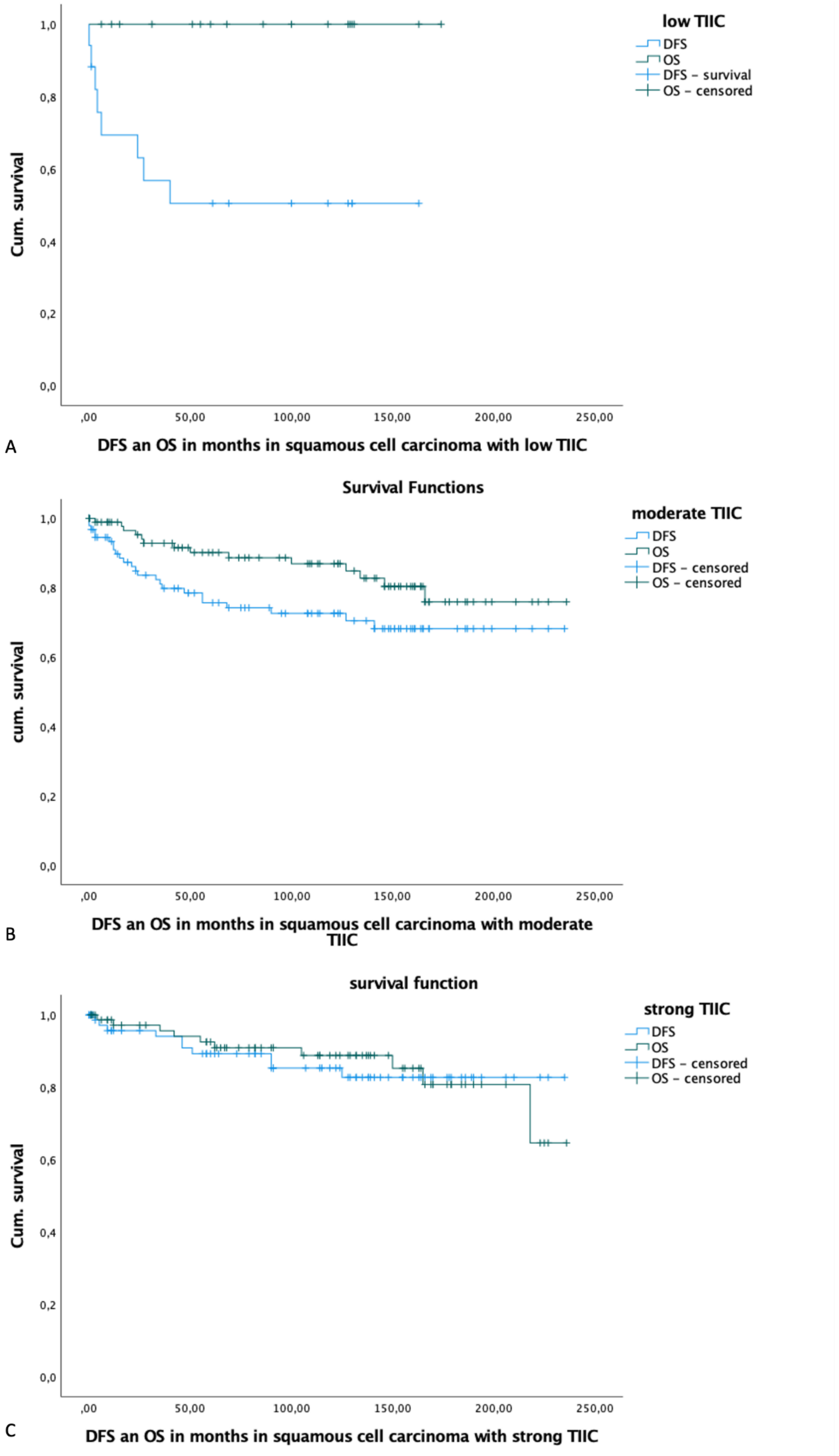

Figure S2: Survival analyses for DFS and OS in CCSCC with low (A), moderate (B) or strong TIIC levels (C).

Parameter		CSCC with low TIIC n = 17		CSCC with moderate TIIC n = 96		CSCC with strong TIIC n=78		
		Mean	Std. dev	Mean	Std. dev.	Mean		p
Age		56.82	15.75	50.39	13.03	47.27	11.44	**.016**
		n	%	n	%	n	%	
pT	pT1	1	5.88	21	21.88	20	25.64	.436
	pT2	5	29.41	19	19.79	13	16.67	
	pT3	11	64.71	56	58.33	45	57.69	
pN	pN1	6	35.29	44	45.83	26	33.33	.227
	pN0	11	64.71	52	54.17	52	66,67	
Grading	G1	1	5.88	3	3.13	8	10.23	.278
	G2	9	52.95	62	64.57	41	52.57	
	G3	6	35.29	28	29.17	27	34.63	
	Missing	1	5.88	3	3.13	2	2.57	
FIGO 2009	1	5	29,41	24	25.0	14	17.95	.175
	2	2	11,77	20	20.83	20	25.64	
	3	5	29.41	18	18.75	5	6.41	
	4	0	0	3	3.13	1	1.28	
	Missing	5	29.41	31	32.29	38	48.72	

Table S1: Comparison for age, pT, pN, Grading and FIGO 2009 in CSCC with different levels of TIIC.
